# Further evaluation of the NWF filter for the purification of *Plasmodium vivax*-infected erythrocytes

**DOI:** 10.1186/s12936-017-1855-3

**Published:** 2017-05-17

**Authors:** Jiangyan Li, Zhiyong Tao, Qian Li, Awtum Brashear, Ying Wang, Hui Xia, Qiang Fang, Liwang Cui

**Affiliations:** 1 Department of Microbiology and Parasitology, Bengbu Medical College, Bengbu, China; 2 Anhui Key Laboratory of Infection and Immunity, Bengbu Medical College, Bengbu, China; 3 Department of Entomology, Pennsylvania State University, 501 ASI Building, University Park, PA USA; 4 Institute of Tropical Medicine, Third Military Medical University, Chongqing, China

**Keywords:** *Plasmodium vivax*, Infected red blood cell, Filter, Purification, Field evaluation

## Abstract

**Background:**

Isolation of *Plasmodium*-infected red blood cells (iRBCs) from clinical blood samples is often required for experiments, such as ex vivo drug assays, in vitro invasion assays and genome sequencing. Current methods for removing white blood cells (WBCs) from malaria-infected blood are time-consuming or costly. A prototype non-woven fabric (NWF) filter was developed for the purification of iRBCs, which showed great efficiency for removing WBCs in a pilot study. Previous work was performed with prototype filters optimized for processing 5–10 mL of blood. With the commercialization of the filters, this study aims to evaluate the efficiency and suitability of the commercial NWF filter for the purification of *Plasmodium vivax*-infected RBCs in smaller volumes of blood and to compare its performance with that of Plasmodipur^®^ filters.

**Methods:**

Forty-three clinical *P. vivax* blood samples taken from symptomatic patients attending malaria clinics at the China–Myanmar border were processed using the NWF filters in a nearby field laboratory. The numbers of WBCs and iRBCs and morphology of *P. vivax* parasites in the blood samples before and after NWF filtration were compared. The viability of *P. vivax* parasites after filtration from 27 blood samples was examined by in vitro short-term culture. In addition, the effectiveness of the NWF filter for removing WBCs was compared with that of the Plasmodipur^®^ filter in six *P. vivax* blood samples.

**Results:**

Filtration of 1–2 mL of *P. vivax*-infected blood with the NWF filter removed 99.68% WBCs. The densities of total iRBCs, ring and trophozoite stages before and after filtration were not significantly different (*P* > 0.05). However, the recovery rates of schizont- and gametocyte-infected RBCs, which were minor parasite stages in the clinical samples, were relatively low. After filtration, the *P. vivax* parasites did not show apparent morphological changes. Culture of 27 *P. vivax*-infected blood samples after filtration showed that parasites successfully matured into the schizont stage. The WBC removal rates and iRBC recovery rates were not significantly different between the NWF and Plasmodipur^®^ filters (*P* > 0.05).

**Conclusions:**

When tested with 1–2 mL of *P. vivax*-infected blood, the NWF filter could effectively remove WBCs and the recovery rates for ring- and trophozoite-iRBCs were high. *P. vivax* parasites after filtration could be successfully cultured in vitro to reach maturity. The performance of the NWF and Plasmodipur^®^ filters for removing WBCs and recovering iRBCs was comparable.

**Electronic supplementary material:**

The online version of this article (doi:10.1186/s12936-017-1855-3) contains supplementary material, which is available to authorized users.

## Background

Malaria is an important vector-borne parasitic disease. According to the World Health Organization estimates, it caused about 0.429 million human deaths in 2015, most of which were due to *Plasmodium falciparum* infections. Outside Africa, *Plasmodium vivax* is the most geographically widespread; more than 50% of malaria cases are caused by this parasite [1]. Compared with falciparum malaria, research on *P. vivax* has lagged behind [1–4]. A major reason for this negligence is the lack of a long-term continuous in vitro culture technique for *P. vivax* [5–7]. As a result, most studies on *P. vivax* rely on infected blood samples obtained from patients. Subsequent experiments often require removal of white blood cells (WBCs) [8–14].

Removing WBCs from blood samples is typically done by density gradient centrifugation or filtration. Density gradient centrifugation is often time-consuming and ineffective, and is often used for purification of WBCs for immunological studies. In comparison, purification of infected red blood cells (iRBCs) mostly involves column filtration using self-made cellulose-based filtration columns or commercial Plasmodipur^®^ filters [15–19]. Cellulose-based filtration columns are cheap, but they need to be installed manually. Despite their simplicity, efficiency, and consistency, Plasmodipur^®^ filters are expensive for resource-limited settings [18–20].

In 2011, Tao et al. [20] developed a non-woven fabric (NWF) filter, and tested the prototype filter for removing WBCs from in vitro cultured *P. falciparum*, rodent malaria parasite *Plasmodium berghei*-infected blood, and 15 clinical samples of *P. vivax*-infected blood. While this laboratory-assembled prototype filter proved to be effective for removing WBCs from malaria-infected blood, Tao et al. did not directly measure the recovery rates of iRBCs, nor did they assess the recovery rates of different developmental stages. In addition, the prototype filter was optimized for processing 5–10 mL of blood sample. Since much smaller volumes (1–2 mL) of blood are often obtained for ex vivo assays of drug sensitivities [16], the performance of the filters for smaller volumes of blood needs to be evaluated. The NWF filters have recently been commercialized and redesigned for better adaptation to syringes (Zhi Xing Bio S&T Co. Ltd, China). Therefore, it is necessary to perform further evaluation of the commercialized filters and compare their performance with the commonly used Plasmodipur^®^ filters. The present study aimed to evaluate the applicability of the NWF filters for purifying *P. vivax*-iRBCs from small volumes of clinical samples.

## Methods

### Sample collection

Forty-three *P. vivax* blood samples were obtained from adult patients with uncomplicated *P. vivax* infections who were attending two malaria clinics near Laiza township in northeast Myanmar and the Nabang township clinic, western Yunnan Province, China, between May and July 2013. Malaria was diagnosed by microscopic examination of thick and thin blood smears. After written informed consent was obtained from the patients, 2–5 mL of venous blood was drawn by trained local nurses into heparin-treated vacutainers. Blood samples were kept at 37 °C in a thermos and immediately transferred to a field laboratory for processing. The field laboratory is located in Nabang township, within 0.5 km of the three clinics. The laboratory was equipped with a light microscope, a centrifuge, an incubator and a candle jar for short-term ex vivo culture of the parasites.

### Purification of *Plasmodium vivax*-infected blood sample by using the NWF filter

The NWF filters redesigned for syringes were purchased from Zhi Xing Bio (ZXBio.net). For purification of iRBCs, 1–2 mL of whole blood was transferred to a 15 mL tube, and centrifuged for 10 min at 2200×*g*. Blood cells were resuspended in two times of the original volume of incomplete McCoy’s 5A medium (Invitrogen, USA) and mixed. The resultant cell suspension was drawn into a 10 mL syringe, which was adapted to the NWF filter inlet interface. The plunger was gently pushed at about 5 mL/min and the flow-through cell suspension was collected into a new 15 mL centrifuge tube. The NWF filter was rinsed with 5 mL McCoy’s 5A medium, and eluted cell suspension was collected in the same tube. The sample was centrifuged for 10 min at 3000 rpm, then the supernatant was discarded, and the pellet was resuspended in McCoy’s 5A medium to a total volume of 5 mL for ex vivo culture purpose.

### Enumeration of WBCs and parasitaemias

Before and after filtration, thin blood smears were stained with Giemsa, and parasitaemias and WBC numbers were enumerated by microscopy. The number of WBCs and iRBCs in 40,000 RBC on the thin films was counted (repeated three times for every sample). The WBC removal rate was calculated as $1 - ({\text{number}}\;{\text{of}}\;{\text{WBCs}}\;{\text{after}}\;{\text{filtration}}/{\text{number}}\;{\text{of}}\;{\text{WBCs}}\;{\text{before}}\;{\text{filtration}}) \times 100\%$. The iRBC recovery rate was calculated as (number of iRBCs after filtration/number of iRBCs before filtration) × 100%.

### Short-term in vitro culture of *Plasmodium vivax* parasites

Of the samples collected, 27 without prior anti-malarial therapy and having >50% of parasites at the ring stage were subjected to short-term in vitro culture. Blood samples were washed three times with McCoy’s 5A medium, resuspended to 2% haematocrit in McCoy’s 5A medium containing 20% human AB^+^ serum from malaria-naive donors. Samples were added into 96-well plates, wherein each well contained 100 μL of the cell suspension. The plates were placed in a candle jar, and incubated at 37 °C for 48 h. Parasite development was examined every 8 h in the first 16 h and every 2 h subsequently. The parasitaemias and morphology of parasite were examined by microscopy of thick and thin smears. This process was repeated three times for each sample.

### Comparison of the NWF and the Plasmodipur^®^ filters

The performance of the NWF filter and Plasmodipur^®^ (EuroProxima, The Netherlands) for removing WBCs and purifying *P. vivax*-iRBCs was compared side by side using six *P. vivax* blood samples. For each blood sample, half was purified with the NWF filter, while the other half was purified with the Plasmodipur^®^ filter per manufacturer’s instructions. The WBC removal rates and iRBC recovery rates of the two different filters were calculated as described above.

### Statistical analysis

All data were analysed using SPSS for Windows v14 (SPSS Inc, USA). Parasite densities were expressed as mean number of iRBCs ± standard error of the mean (SEM) in 40,000 RBCs. Statistical significance was set at *P* ≤ 0.05. Paired *t* tests were used to compare the WBC removal rates and iRBC recovery rates of the two filters. Mann–Whitney U tests were used to compare the iRBC density of samples before and after NWF filter filtration. Spearman’s correlation coefficient was used for the analysis between WBC removal rates and the initial WBC counts. Unpaired *t* tests were used to compare WBC removal rates of samples with different parasitaemias by the NWF filter.

### Ethical considerations

The study involving collection of venous blood from malaria patients was approved by the institutional review board of Pennsylvania State University (#34319).

## Results

### Removal of WBCs and recovery of parasites from *Plasmodium vivax*-infected blood

**Fig. 1:**
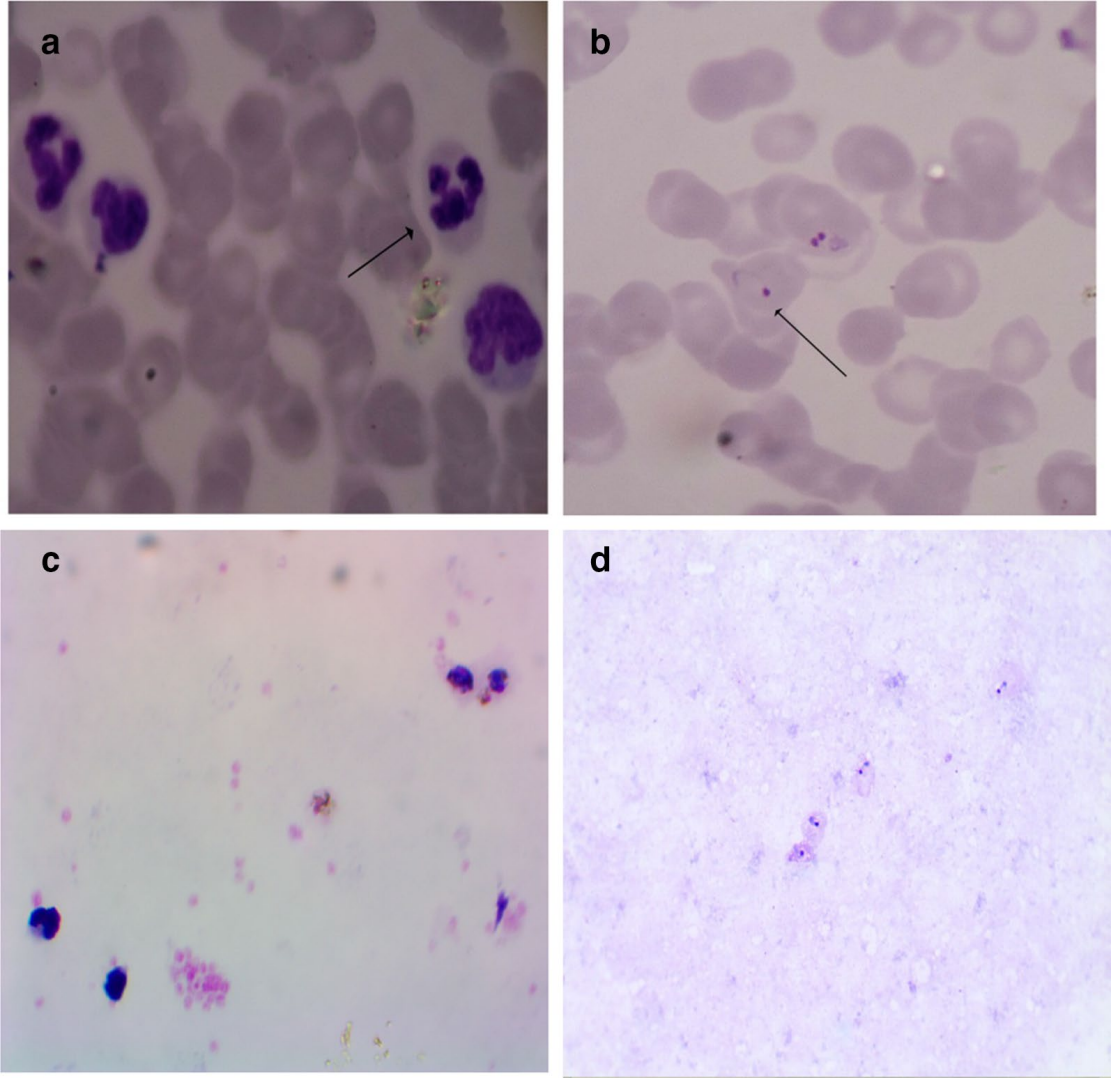
Effective removal of white blood cells by the non-woven fabric filter. Shown here are representative thin blood smear before filtration (**a**) and after filtration (**b**), as well as representative thick blood smear before filtration (**c**) and after filtration (**d**). The *arrow* in **a** indicates a white blood cell, while the *arrow* in **b** marks a *P. vivax*-infected red blood cell

White blood cell removal and parasite recovery were tested with the NWF filters in 43 *P. vivax*-infected whole blood samples collected from patients attending malaria clinics. The average parasitaemia was 0.25%, and most parasites were at the immature asexual stages, which contributed to 89.4% of the total parasites (67.9% rings and 21.5% trophozoites), whereas schizonts and gametocytes constituted 8.2 and 2.5%, respectively (Table 1; Additional file 1: Table S1). Filtration with the NWF filters removed 99.67% WBCs (range 98.00–100%) (Fig. 1).

**Table 1:** Summary of parasite stages of the 43 clinical samples

Parasite stages	Number of iRBCs/40,000 RBCs Mean (STD)	Proportion (%)
Rings	67.42 (98.78)	67.91
Trophozoites	21.33 (37.43)	21.49
Schizonts	8.09 (9.94)	8.15
Gametocytes	2.43 (3.30)	2.45
All stages	99.28 (108.73)	100

**Fig. 2:**
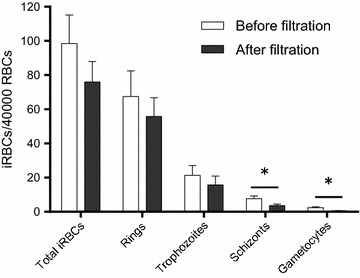
Comparison of the densities of infected red blood cells before and after filtration by the NWF filter. *Asterisk* indicates significant difference at *P* < 0.05 (Mann–Whitney U test)

Filtration with the NWF filters did not lead to obvious morphological changes of the different stages of *P. vivax* (ring, trophozoite, schizont, gametocyte) in the 43 samples tested. Although the overall densities of iRBCs before filtration (98.6 ± 16.6 iRBCs/40,000 RBCs) and after filtration (75.9 ± 12.1 iRBCs/40,000 RBCs) were not significantly different (Figs. 1, 2), losses of the iRBCs were observed. The mean recovery rate of all iRBCs was 82.76% and it varied between *P. vivax* stages. The recovery rates for ring-, trophozoite-, schizont-, and gametocyte-iRBCs were 88.92, 76.83, 61.71, and 57.77%, respectively. The densities of ring- and trophozoite-iRBCs before (67.4 ± 15.1 and 21.3 ± 5.7 iRBCs/40,000 RBCs, respectively) and after filtration (55.8 ± 11.0 and 15.7 ± 5.2 iRBCs/40,000 RBCs, respectively) with the NWF filter were not significantly different (*P* > 0.05, *t* test, Fig. 2). However, for schizont- and gametocyte-iRBCs, which were the minor parasite populations in the infected blood (Table 1), the recovery rates were relatively low, and the parasite densities of these stages after filtration (3.7 ± 0.8 and 0.6 ± 0.2 iRBCs/40,000 RBCs, respectively) were significantly lower than those before filtration (7.7 ± 1.5 and 2.5 ± 0.5 iRBCs/40,000 RBCs, respectively) (*P* < 0.05, *t* test, Fig. 2). Additionally, there was a modest, yet significant, negative correlation between WBC removal rates and the initial WBC counts, with the Spearman’s correlation coefficient of −0.474 (*P* < 0.001, Table 2).

**Table 2:** Correlation between white blood cell removal rates and initial white blood cell counts

WBC counts before filtration (WBCs per 40,000 RBCs)	N	Number of samples with different WBC removal rates after filtration			
		100%	99.00–99.99%	98.00–98.99%	97.00–97.99%
<100	33	27	0	4	2
100–200	8	5	2	1	0
>200	2	0	2	0	0
Total number of samples	43	32	4	5	2

### Short-term in vitro culture of filtrated *Plasmodium vivax* samples

**Fig. 3:**
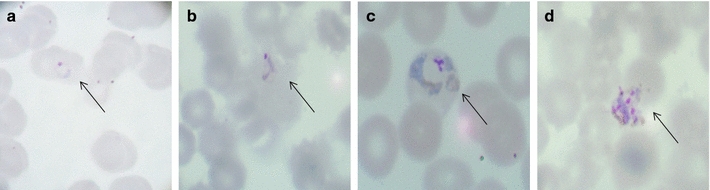
Short-term in vitro culture of *Plasmodium vivax* after purification by the non-woven fabric filter to show ring (**a**), trophozoites (**b**, **c**), and schizont (**d**). *Arrows* point to the parasites

To test whether filtration by the NWF filters affect the culture performance of the *P. vivax*-infected blood samples, filtrated parasites were subjected to short-term in vitro culture. After 24–36 h culture of the ring-stage iRBCs, parasites could mature into the schizont stage (Fig. 3).

### Comparison of the NWF filter with the Plasmodipur^®^ filter

**Fig. 4:**
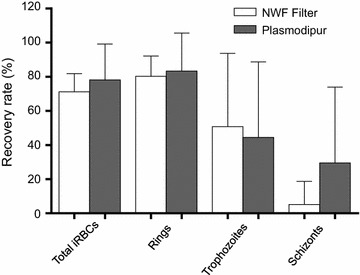
Comparison of infected red blood cell recovery rates after filtration by the non-woven fabric filter and Plasmodipur^®^ filter

Six blood samples from *P. vivax* patients were used for side-by-side comparison of the two filters. The mean WBC removal rates of the two filters were not significantly different (100% for the NWF filters, and 95.9–100% for Plasmodipur^®^ filters; *P* > 0.05). Furthermore, the recovery rates of total iRBC and different stages (ring, trophozoite, schizont) purified with the two filters were not significantly different (57.1–84.0% for the NWF filters, and 41.4–98.6% for Plasmodipur^®^ filters; *P* > 0.05, Fig. 4). Though Plasmodipur^®^ performed better than the NWF filter with some samples in the schizont recovery rate, there were large variations between samples and the overall recovery rates by the two filters were not significantly different (Fig. 4). Because the gametocyte densities of the blood samples were too low, the difference in gametocyte recovery rate between the two filters could not be compared.

## Discussion

Removing WBCs from *P. vivax*-infected blood to obtain purified parasites is essential for numerous in vitro studies of the malaria parasites such as in vitro culture, drug susceptibility analysis, genomics, and proteomics [8, 10–12, 14, 21]. The effectiveness of removing WBCs from samples will directly affect the results of these studies, possibly determining their success or failure. Currently, the main methods for removing WBCs from malaria-infected blood are Plasmodipur^®^ filter filtration and cellulose-based filtration methods such as CF11 columns. These two methods can achieve satisfactory WBC removal. However, cellulose-based filtration columns need to be installed manually. Plasmodipur^®^ filters, on the other hand, are simple and effective but often expensive in malaria-endemic countries. The commercialized NWF filter is priced at 100 CNY. It also can remove WBCs from *Plasmodium*-infected blood rapidly and effectively. In this evaluation, the WBC removal rate by the NWF filter was as high as 99.67%. Morphology of different stages of *P. vivax* after filtration with the NWF filter showed no apparent changes. These findings indicate that *P. vivax* parasites can be purified from clinical samples with this NWF filter without incurring damage and can be used for subsequent research.

Whereas Tao et al. found that the recovery rate of total RBCs were 95.48%, they did not measure the recovery rates of iRBCs and different parasite stages, which should be important parameters for determining the performance of the filters. This study found that ring- and trophozoite-iRBCs after filtration had satisfactorily high recovery rates, and parasite densities before and after filtration were not significantly different. However, the recovery rates for schizonts and gametocytes were relatively modest and parasite densities after filtration were significantly lower than those before filtration, suggesting that these parasite stages might have been intercepted at higher rates. This result suggests that the NWF filter is more suitable for purifying immature parasites from *P. vivax*-infected blood samples. It is noteworthy that both schizonts and gametocytes were minor parasite stages in the samples studied. Future studies might be needed to evaluate this filter for samples with higher densities of schizonts and gametocytes. In a small set of samples, the performance of this filter was comparable with that of Plasmodipur^®^ in removing WBCs and recovering iRBCs. Future comparison of gametocyte-rich samples may be needed to test the recovery rates of gametocytes.

## Conclusions

This study evaluated the commercial NWF filters for removing WBCs and purifying iRBCs in 43 clinical *P. vivax* samples. Though the prototype NWF filters were optimized for processing 5–10 mL of blood, the commercial NWF filters also showed effective removal of WBCs and recovery of total iRBCs with 1–2 mL of infected blood. The recovery rates of iRBCs containing earlier asexual erythrocytic stages (rings and trophozoites) were high, whereas the recovery rates of schizont- and gametocyte-iRBCs were relatively low. The overall performance of the NWF filter and Plasmodipur^®^ for removing WBCs and recovering iRBCs were comparable; the latter appeared to perform better in recovering schizont- and gametocyte-infected RBCs in some samples.

## Additional file

**Additional file 1: Table S1.** Parasite stages of the 43 P. vivax clinical samples.

## Abbreviations

NWF: non-woven fabric
iRBC: infected red blood cell
WBC: white blood cell
