# Supplementary material for: Further evaluation of the NWF filter for the purification of Plasmodium vivax-infected erythrocytes
Source: Malar J. 2017 May 17;16:201. doi: 10.1186/s12936-017-1855-3 (PMC5436455; doi:10.1186/s12936-017-1855-3)
Supplement: Supplementary file 1 — Additional file 1: Table S1. Parasite stages of the 43 P. vivax clinical samples. [file 12936_2017_1855_MOESM1_ESM.pdf]

Table S1. Parasite stages of the 43 *P. vivax* clinical samples.

	Rings		Trophozoites		Schizonts		Gametocytes		All stages
	Numbers	%	Numbers	%	Numbers	%	Numbers	%	Numbers
1	66	70.21	11	11.70	7	7.45	10	10.64	94
2	4	80.00	1	20.00	0	0.00	0	0.00	5
3	2	100.00	0	0.00	0	0.00	0	0.00	2
4	1	5.00	12	60.00	7	35.00	0	0.00	20
5	1	9.09	8	72.73	2	18.18	0	0.00	11
6	63	73.26	11	12.79	8	9.30	4	4.65	86
7	16	94.12	1	5.88	0	0.00	0	0.00	17
8	34	85.00	4	10.00	2	5.00	0	0.00	40
9	38	74.51	9	17.65	3	5.88	1	1.96	51
10	15	40.54	15	40.54	3	8.11	4	10.81	37
11	14	48.28	3	10.34	12	41.38	0	0.00	29
12	19	76.00	2	8.00	4	16.00	0	0.00	25
13	10	21.28	10	21.28	25	53.19	2	4.26	47
14	20	90.91	2	9.09	0	0.00	0	0.00	22
15	3	5.08	6	10.17	35	59.32	15	25.42	59
16	14	36.84	12	31.58	10	26.32	2	5.26	38
17	23	67.65	8	23.53	2	5.88	1	2.94	34
18	62	77.50	12	15.00	6	7.50	0	0.00	80
19	6	11.32	31	58.49	12	22.64	4	7.55	53
20	1	3.70	5	18.52	21	77.78	0	0.00	27
21	152	92.68	5	3.05	3	1.83	4	2.44	164
22	42	100.00	0	0.00	0	0.00	0	0.00	42
23	360	77.09	63	13.49	37	7.92	7	1.50	467
24	0	0.00	210	92.11	10	4.39	8	3.51	228
25	83	39.71	88	42.11	31	14.83	7	3.35	209
26	16	37.21	21	48.84	1	2.33	5	11.63	43
27	69	81.18	8	9.41	6	7.06	2	2.35	85
28	0	0.00	28	77.78	7	19.44	1	2.78	36
29	27	87.10	3	9.68	1	3.23	0	0.00	31
30	44	86.27	5	9.80	2	3.92	0	0.00	51
31	243	98.38	2	0.81	2	0.81	0	0.00	247
32	124	87.32	14	9.86	1	0.70	3	2.11	142
33	108	83.08	10	7.69	5	3.85	7	5.38	130
34	38	79.17	8	16.67	2	4.17	0	0.00	48
35	160	96.39	3	1.81	3	1.81	0	0.00	166
36	45	56.25	18	22.50	14	17.50	3	3.75	80
37	400	99.75	0	0.00	1	0.25	0	0.00	401
38	354	91.47	23	5.94	8	2.07	2	0.52	387
39	123	54.42	65	28.76	33	14.60	5	2.21	226
40	43	74.14	9	15.52	4	6.90	2	3.51	58
41	45	72.58	8	12.90	9	14.52	0	0.00	62
42	7	6.73	91	87.50	4	3.85	2	1.92	104
43	4	4.65	71	82.56	6	6.98	5	5.81	86
